# Intraoperative dexmedetomidine use associated with prolonged postanesthesia care unit recovery time after lung resection: a retrospective cohort study

**DOI:** 10.3389/fphar.2025.1649139

**Published:** 2025-11-26

**Authors:** Zhenglian Gao, Yan Jiang, Yixin Liu, Chang Yang, Bangjian Zhang, Ming Li

**Affiliations:** 1 Department of Anesthesiology, Panzhihua Central Hospital, Panzhihua, Sichuan, China; 2 Department of Nuclear Medicine, Panzhihua Central Hospital, Panzhihua, Sichuan, China

**Keywords:** dexmedetomidine, postanesthesia care unit, delayed recovery, risk factor, lung resection

## Abstract

**Background:**

Dexmedetomidine, despite its wide-ranging benefits, may also pose a risk of delayed recovery in the postanesthesia care unit (PACU). Few studies have examined the relationship between dexmedetomidine and delayed recovery in the PACU after lung resection. Therefore, this retrospective cohort study aimed to investigate the effect of dexmedetomidine on PACU recovery time.

**Methods:**

This study identified 1,397 eligible patients [dexmedetomidine-free (NO-DEX) group, n = 638; intraoperative dexmedetomidine use (DEX) group, n = 759] among 1,980 patients undergoing lung resection from January 2020 to December 2023. The primary outcome was the relationship between dexmedetomidine exposure and the risk of prolonged PACU recovery time; secondary outcomes were independent risk factors affecting PACU recovery time. The data were analyzed using propensity score matching and univariate and multivariate logistic regression analyses, as appropriate. In addition, we also developed a nomogram, which was evaluated using the calibration curve, receiver operating characteristic (ROC) curve, and decision curve analysis (DCA).

**Results:**

After propensity score matching, there were 521 patients in each group. The incidence of PACU recovery time exceeding 60 min was 25.48%. Univariate logistic regression analysis showed that intraoperative dexmedetomidine use was associated with the risk of prolonged PACU recovery time [odds ratio (OR): 1.58; 95% confidence interval (CI): 1.18–2.11; *p* = 0.002]. Multivariate logistic regression analysis also showed a significant difference in the risk of prolonged PACU recovery time (OR: 1.60; 95% CI: 1.19–2.15; *p* = 0.002) between the NO-DEX and DEX groups. Sensitivity analyses under varying assumptions confirmed the robustness of our primary results. Independent risk factors for delayed recovery in the PACU included intraoperative dexmedetomidine use, advanced age, smoking, cardiovascular disease, and ASA physical status III, whereas regional block was associated with reduced risk. The predictive nomogram demonstrated moderate discrimination and estimated a 70% probability of delayed PACU recovery in patients presenting with all identified risk factors.

**Conclusion:**

Our data demonstrated that intraoperative dexmedetomidine use was associated with prolonged PACU recovery time following lung resection, potentially increasing the pressure on busy recovery rooms.

## Introduction

The recovery unit, also known as the postanesthesia care unit (PACU), is a place where patients gradually recover from the stress state of anesthesia and surgery, and it provides ongoing medical care by professionally trained anesthesia-related personnel until the patients have completely emerged from anesthesia (Wehner and German Society for Anesthesiology and Intensive Care Medicine and Professional Association of German Anesthetists, 2010; Zemedkun et al., 2022). The PACU plays a pivotal role in ensuring perioperative patient safety. Strategic patient management within the PACU enhances operating room workflow efficiency, with particular relevance for lung resection patients who demonstrate prolonged recovery trajectories and extended PACU length of stay (Ziarnik and Grogan, 2015; Puccinelli et al., 2020). Extended PACU stays impose a substantial burden on healthcare resources, may compromise operating room efficiency, and correlate with elevated healthcare costs and increased incidence of short-term complications (Fairley et al., 2019; Mann-Farrar et al., 2019). Additionally, PACU length of stay, a critical indicator of postoperative recovery quality, may be directly influenced by variations in analgesic and anesthetic techniques (Maheshwari et al., 2020; Schulz et al., 2020; Zhang et al., 2022; Sprung et al., 2023).

Dexmedetomidine, a highly selective α_2_-adrenoreceptor agonist, is widely administered as an adjunct to anesthesia for both surgery and procedural sedation. Dexmedetomidine possesses several advantageous characteristics, including sedation, anxiolysis, anti-inflammatory effects, sympathetic inhibition, bronchodilation, and tachycardia prevention (Hoy and Keating, 2011; Keating, 2015). In addition, dexmedetomidine can reduce respiratory depression compared to other sedative drugs (Zhuang et al., 2011), decrease postanesthetic delirium (Duan et al., 2018), and diminish postoperative opioid requirements (Grape et al., 2019). The reduction in respiratory depression and nausea/vomiting has prompted the inclusion of dexmedetomidine into Enhanced Recovery After Surgery (ERAS) protocols at numerous institutions. In particular, in bariatric and cardiac surgery, dexmedetomidine has been found to accelerate recovery. Although dexmedetomidine enhances awakening and extubation conditions (Tung et al., 2020), its pharmacokinetic profile (half-life: 2–3 h) may contribute to prolonged sedation and subsequent delays in hospital discharge following sedative colonoscopy (Edokpolo et al., 2019). For example, dexmedetomidine delays recovery and prolongs discharge times by up to 101 min compared to propofol when used for sedation during magnetic resonance imaging and non-invasive procedures (Heard et al., 2008; Phelps et al., 2015). Nevertheless, existing evidence regarding the effects of dexmedetomidine on early postoperative recovery is insufficient to justify changes in current clinical practice. Well-designed clinical studies are, therefore, urgently warranted to establish whether intraoperative dexmedetomidine administration prolongs recovery processes, particularly the duration of the PACU stay, following lung resection.

Thus, we carried out a retrospective cohort study to assess whether the intraoperative use of dexmedetomidine influences the PACU recovery time after lung resection. Other risk factors related to the prolonged PACU recovery time were also analyzed in this study.

## Methods

### Study design and population

This retrospective cohort study followed the guidelines of the Declaration of Helsinki and was approved by the Ethics Committee of Panzhihua Central Hospital on 04 Jan 2023 (No. PZHZXYY-REC-003). Data were fully anonymized before analysis. The requirement for informed consent was waived because the study was based on an anonymous analysis of the retrospective electronic medical records.

We retrospectively reviewed the electronic medical records of all patients who underwent lung resection at Panzhihua Central Hospital from January 2020 to December 2023. Exclusion criteria included emergency surgery, age <18 years, direct transfer to destinations other than the PACU postoperatively, American Society of Anesthesiologists (ASA) physical status ≥ IV, and incomplete medical records. The reasons for patients being transferred directly to the ICU or ward, bypassing the PACU, were as follows: (1) preoperative impaired consciousness; (2) extremely poor physical condition; and (3) intraoperative complications, including massive hemorrhage, anaphylactic shock, or severe subcutaneous emphysema. The postoperative decision to transfer patients to the ICU or ward was not influenced by dexmedetomidine use.

### Anesthetic technique for lung resection

The anesthetic technique for lung resection followed the institutional guidelines of Panzhihua Central Hospital. All patients underwent general anesthesia with double-lumen endobronchial intubation. Standard monitoring included electrocardiography, blood pressure, pulse oximetry, end-tidal carbon dioxide, temperature, and, when indicated, invasive arterial and central venous pressure monitoring. Anesthesia was induced with propofol, etomidate, sufentanil, midazolam, and rocuronium and maintained with either total intravenous anesthesia using propofol and remifentanil or inhalational anesthesia with sevoflurane and remifentanil. A selective preoperative thoracic paravertebral or epidural block was applied for regional anesthesia. All patients received patient-controlled intravenous analgesia with a total of 150 mL of sufentanil (1.0 μg/mL) for postoperative pain control. Patients were stratified into two groups based on intraoperative dexmedetomidine administration: the dexmedetomidine group (DEX group) received intravenous infusion of dexmedetomidine during surgery, while the control group (NO-DEX group) did not receive dexmedetomidine. The institutional protocol for dexmedetomidine administration is as follows: a loading dose of 1 μg/kg is administered within 10 min prior to anesthetic induction, followed by maintenance infusion at 0.1–0.5 μg/(kgh) titrated according to patient hemodynamics, with cessation 30–60 min before surgical completion.

### Measurements

We recorded the following data from the electronic medical records: age, sex, body mass index (BMI), smoking status, drinking status, comorbidities at admission (hypertension, diabetes, cardiovascular disease, cerebrovascular diseases, or respiratory diseases), ASA physical status, duration of anesthesia, duration of one-lung ventilation, anesthesia method, use of regional blocks, intraoperative use of various drugs (dexmedetomidine, vasoactive drugs, or opioids), intraoperative hypoxemia, perioperative blood transfusion, infusion volume, urine volume, blood loss volume, surgical site, the type of operation (segmental/wedge resection, lobectomy, or pneumonectomy), video-assisted thoracic surgery (VATS) or thoracotomy, and the PACU recovery time. All patients in this study received opioids. Total opioid consumption was converted to intravenous morphine equivalent. Dexmedetomidine doses were extracted from anesthesia records and converted to μg/kg based on patient body weight.

### Study outcome

The primary outcome was the association between the dexmedetomidine exposure and the risk of prolonged PACU recovery time. The secondary outcomes were the independent risk factors for affecting the PACU recovery time. The PACU recovery time was defined as the period from entering the PACU to leaving the PACU. The delayed recovery was defined as a PACU recovery time of more than 60 min after entering the PACU. Discharge criteria from the PACU for patients under general anesthesia at our institution were as follows: Steward score ≥4 points; Aldrete score ≥9 points. Patients who exhibited hemodynamic instability or respiratory complications requiring continued respiratory support or intensive monitoring were transferred to the ICU and therefore excluded from the analysis.

### Statistical analysis

The sample size calculation was not performed in advance because this retrospective study included all patients who underwent lung resection at Panzhihua Central Hospital between January 2020 and December 2023. Therefore, the analysis was conducted using the available patient data within this period.

The study results were described as the number with percentage for categorical variables and as the mean with standard deviation or the median with interquartile range for continuous variables, as appropriate. The Shapiro–Wilk test was used to assess the normality of the distribution of continuous variables. The differences between groups of continuous variables were compared using the independent-sample t-test or Wilcoxon rank-sum test, and categorical variables were compared using the chi-square test.

Propensity score matching was used to reduce the potential confounding effects of variables and the differences in baseline characteristics between the two groups. Logistic regression analysis was used to estimate the propensity score of patients in the DEX group. Two groups of patients were matched at a ratio of 1:1 using the nearest neighbor method, with a caliper of 0.1 and a random seed number of 123. The variables used for matching were age, sex, BMI, smoking status, drinking status, hypertension, diabetes, cardiovascular disease, cerebrovascular diseases, respiratory diseases, ASA physical status, duration of anesthesia, duration of one-lung ventilation, anesthesia method, regional blocks, morphine equivalent, vasoactive drugs, intraoperative hypoxemia, perioperative blood transfusion, infusion volume, urine volume, blood loss volume, surgical site, type of operation, and VATS. The standardized mean difference (SMD) was used to evaluate the balance on baseline characteristics between the two groups, with a value <0.10 indicating good balance.

In the propensity-matched cohort, univariate and multivariate logistic regression analyses were used to evaluate the association between dexmedetomidine and PACU recovery time after general anesthesia. In the total study cohort, univariate and multivariate logistic regression analyses were performed to explore the independent risk factors influencing PACU recovery time in patients undergoing lung resection. Results were expressed as odds ratio (OR) and 95% confidence interval (CI). Variables with *p*-values <0.05 in univariate logistic regression analysis were included in the multivariate logistic regression analysis. Variables with *p* < 0.05 in the multivariate regression analysis were used to develop a nomogram, which was evaluated using the calibration curve, receiver operating characteristic (ROC) curve, and decision curve analysis (DCA).

In addition, to assess the robustness of our findings, we performed the following sensitivity analyses: Model A incorporated variables with *p*-values <0.05 in univariate logistic regression analysis and those with clinical significance, whereas Model B incorporated all variables. Furthermore, the total study cohort prior to propensity score matching was analyzed to further assess the robustness of our findings.

All analyses were performed using R software version 4.4.2 (R Foundation for Statistical Computing, Austria). A two-tailed *p* < 0.05 was considered statistically significant.

## Results

**FIGURE 1:**
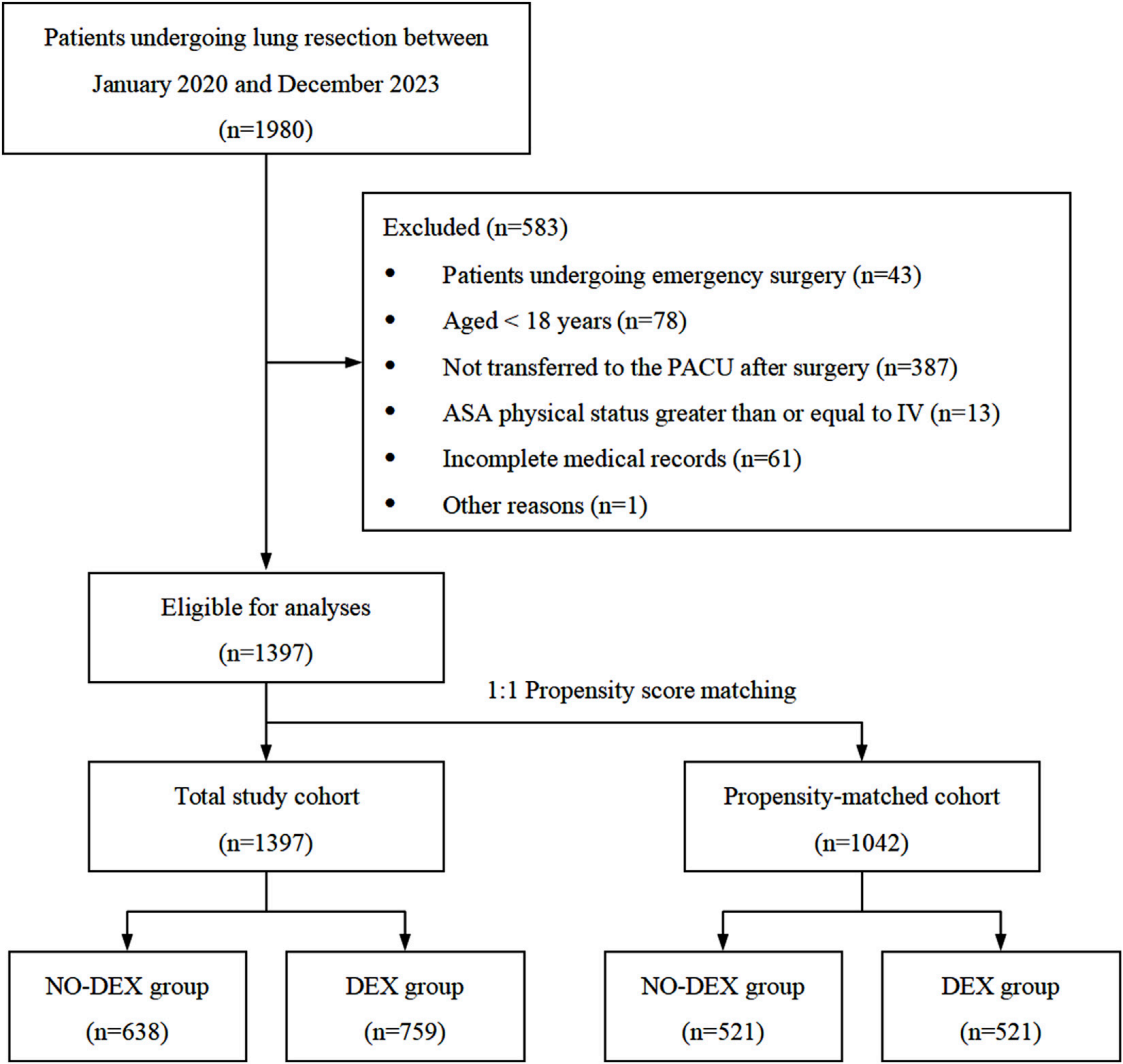
Flow diagram of the study population.

In total, 1,397 patients (NO-DEX group, n = 638; DEX group, n = 759) were finally included in the analyses (Figure 1). A total of 356 patients (25.48%) had PACU recovery times exceeding 60 min.

**FIGURE 2:**
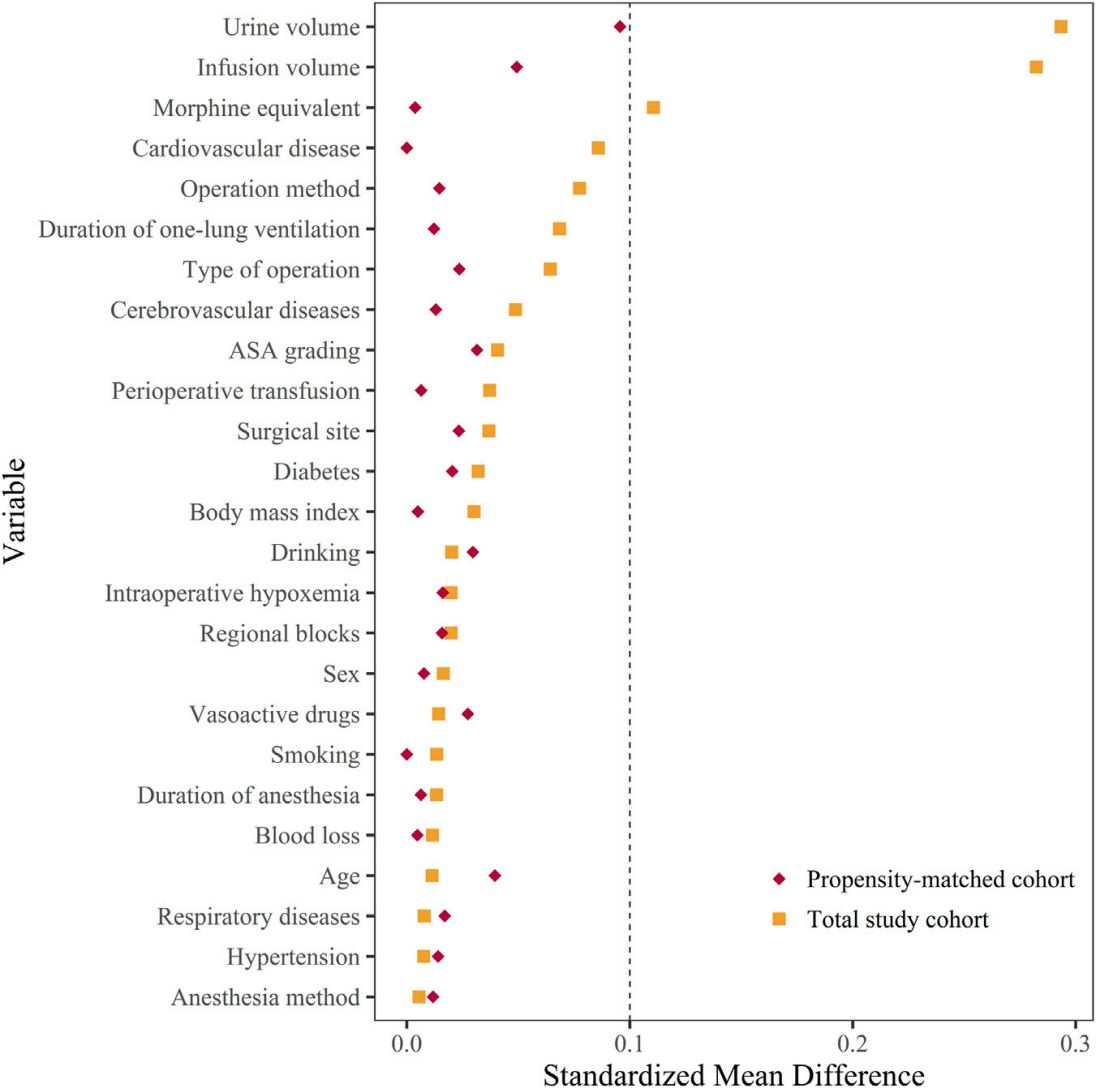
Distribution of standardized mean difference for variables included before and after matching. Standardized mean difference values <0.10 presented that the variable was well matched and that there was no difference between the two groups.

**FIGURE 3:**
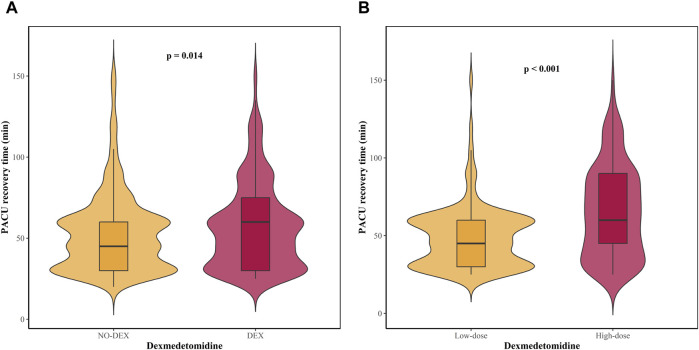
Association between dexmedetomidine doses and PACU recovery time. **(A)** Not received dexmedetomidine vs. received dexmedetomidine. **(B)** Received cumulative doses above the median (1.60 μg/kg) vs. received cumulative doses below the median.

The baseline characteristics of patients in both groups are presented for the total study cohort and the propensity score-matched cohort (Table 1). In the total study cohort, there were differences between the two groups in intraoperative infusion volume, urine volume, and morphine equivalent (SMD ≥0.10). After propensity score matching, there were 521 patients in each group. The SMD suggested that all variables were well matched between the two groups (SMD <0.10) (Figure 2). The median PACU recovery time was 50 (30, 65) minutes in the total study cohort and 50 (30, 60) minutes in the propensity-matched cohort. Statistically significant differences in the PACU recovery time were observed between the DEX and NO-DEX groups in both cohorts (Table 2). Patients receiving dexmedetomidine had significantly longer PACU stays than those without dexmedetomidine exposure (60 [30, 75] vs. 45 [30, 60] minutes, *p* = 0.014; Figure 3A). This effect was more pronounced in patients receiving cumulative doses above the median threshold of 1.60 μg/kg (60 [45, 90] vs. 45 [30, 60] minutes, *p* < 0.001; Figure 3B).

**TABLE 1:** Patient characteristics before and after matching.

Variable	Total study cohort			Propensity-matched cohort^a^		
	NO-DEX	DEX	*SMD* ^b^	NO-DEX	DEX	*SMD*
	(n = 638)	(n = 759)		(n = 521)	(n = 521)	
Age (years)	58.27 ± 8.20	58.17 ± 8.33	0.011	58.05 ± 8.42	58.37 ± 8.19	0.040
Sex (male)	349 (54.7)	409 (53.9)	0.016	284 (54.5)	286 (54.9)	0.008
BMI^c^ (kg/m^2^)	23.48 ± 3.13	23.57 ± 3.06	0.030	23.48 ± 3.09	23.46 ± 3.12	0.005
Smoking	269 (42.2)	315 (41.5)	0.013	220 (42.2)	220 (42.2)	<0.001
Drinking	184 (28.8)	212 (27.9)	0.020	154 (29.6)	147 (28.2)	0.030
Comorbidities at admission						
Hypertension	139 (21.8)	163 (21.5)	0.008	113 (21.7)	110 (21.1)	0.014
Diabetes	64 (10.0)	69 (9.1)	0.032	47 (9.0)	44 (8.4)	0.020
Cardiovascular disease	85 (13.3)	80 (10.5)	0.086	58 (11.1)	58 (11.1)	<0.001
Cerebrovascular diseases	58 (9.1)	80 (10.5)	0.049	49 (9.4)	51 (9.8)	0.013
Respiratory diseases	35 (5.5)	43 (5.7)	0.008	29 (5.6)	27 (5.2)	0.017
ASA^c^ grading			0.041			0.031
Ⅰ	179 (28.1)	214 (28.2)		142 (27.3)	138 (26.5)	
Ⅱ	362 (56.7)	419 (55.2)		290 (55.7)	298 (57.2)	
Ⅲ	97 (15.2)	126 (16.6)		89 (17.1)	85 (16.3)	
Anesthesia method			0.006			0.012
INHA^c^	390 (61.1)	466 (61.4)		316 (60.7)	313 (60.1)	
TIVA^c^	248 (38.9)	293 (38.6)		205 (39.3)	208 (39.9)	
Regional blocks	246 (38.6)	300 (39.5)	0.020	200 (38.4)	204 (39.2)	0.016
Duration of anesthesia (hour)	3.54 ± 0.94	3.53 ± 0.92	0.013	3.50 ± 0.91	3.50 ± 0.93	0.006
Duration of one-lung ventilation (hour)	2.05 ± 0.86	1.99 ± 0.82	0.068	1.99 ± 0.83	1.98 ± 0.82	0.012
Morphine equivalent (mg)	119.71 ± 43.50	115.05 ± 40.76	0.111	115.84 ± 39.79	115.69 ± 42.35	0.004
Vasoactive drugs	260 (40.8)	304 (40.1)	0.014	208 (39.9)	215 (41.3)	0.027
Intraoperative hypoxemia	9 (1.4)	9 (1.2)	0.020	7 (1.3)	8 (1.5)	0.016
Perioperative transfusion	65 (10.2)	69 (9.1)	0.037	52 (10.0)	51 (9.8)	0.006
Infusion volume (mL)	1704.31 ± 485.93	1844.33 ± 505.78	0.282	1755.76 ± 474.95	1780.33 ± 520.48	0.049
Urine volume (mL)	338.46 ± 200.91	396.67 ± 195.80	0.293	347.10 ± 199.57	365.30 ± 180.43	0.096
Blood loss (mL)	97.84 ± 81.26	96.63 ± 123.95	0.012	96.76 ± 80.37	97.22 ± 112.52	0.005
Surgical site (left)	284 (44.5)	324 (42.7)	0.037	222 (42.6)	216 (41.5)	0.023
Type of operation			0.064			0.024
Segmental/wedge resection	194 (30.4)	209 (27.5)		151 (29.0)	151 (29.0)	
Lobectomy	439 (68.8)	543 (71.5)		366 (70.2)	367 (70.4)	
Pneumonectomy	5 (0.8)	7 (0.9)		4 (0.8)	3 (0.6)	
VATS^c^	497 (77.9)	615 (81.0)	0.077	422 (81.0)	419 (80.4)	0.015

Data are presented as the mean ± SD or n (%).

^a^ Logistic regression analysis was used to estimate the propensity score of patients in the DEX group including all variables. The two groups of patients were matched at a ratio of 1:1 by the nearest neighbor method with a caliper of 0.1.

^b^ SMD, standardized mean difference. SMD <0.10 presented that the variable was well matched and that there was no difference between the two groups.

^c^ ASA, American Society of Anesthesiologists; BMI, body mass index; INHA, inhalational anesthesia; TIVA, total intravenous anesthesia; VATS: video-assisted thoracic surgery.

**TABLE 2:** PACU recovery time.

PACU recovery time (min)	Total		NO-DEX group		DEX group		*p*-value
	n	Median (interquartile range)	n	Median (interquartile range)	n	Median (interquartile range)	
Total study cohort	1,397	50 (30, 65)	638	45 (30, 60)	759	60 (30, 75)	0.001
Propensity-matched cohort	1,042	50 (30, 60)	521	45 (30, 60)	521	60 (30, 75)	0.014

The univariate and multivariate logistic regression analysis models were constructed to compare the association between dexmedetomidine and the risk of prolonged PACU recovery time in the propensity-matched cohort (Table 3). Univariate logistic regression analysis demonstrated that intraoperative dexmedetomidine use was significantly associated with the risk of prolonged PACU recovery time (OR: 1.58; 95% CI: 1.18–2.11; *p* = 0.002). Variables with *p*-values <0.05 in univariate logistic regression analysis were subsequently entered into the multivariate logistic regression model. After adjustment for confounding variables, intraoperative dexmedetomidine use remained independently associated with the risk of prolonged PACU recovery time (OR: 1.60; 95% CI: 1.19–2.15; *p* = 0.002). Sensitivity analyses confirmed the robustness of our primary findings across different model specifications: Model A (OR: 1.60; 95% CI: 1.19–2.16; *p* = 0.002), Model B (OR: 1.61; 95% CI: 1.19–2.18; *p* = 0.002), and the total study cohort model (OR: 1.73; 95% CI: 1.34–2.23; *p* < 0.001).

**TABLE 3:** Univariate and multivariate logistic regression analyses for PACU recovery time after propensity score matching.

Variable	Univariate logistic regression analysis		Multivariate logistic regression analysis	
	Odds ratio [95% CI^a^]	*p*-value	Odds ratio [95% CI^a^]	*p*-value
Dexmedetomidine				
NO-DEX	Reference		Reference	
DEX	1.58 [1.18–2.11]	0.002	1.60 [1.19, 2.15]	0.002
Age (years)	1.03 [1.01–1.05]	0.002	1.02 [1.00, 1.04]	0.028
Sex (male)	0.84 [0.63–1.12]	0.231		
BMI^a^ (kg/m^2^)	0.99 [0.95–1.04]	0.691		
Smoking	1.60 [1.20–2.13]	0.001	1.57 [1.17, 2.11]	0.003
Drinking	0.98 [0.71–1.34]	0.882		
Comorbidities at admission				
Hypertension	0.99 [0.70–1.40]	0.959		
Diabetes	1.02 [0.62–1.68]	0.948		
Cardiovascular disease	1.58 [1.04–2.40]	0.033	1.46 [0.93, 2.26]	0.098
Cerebrovascular diseases	0.83 [0.50–1.38]	0.476		
Respiratory diseases	1.18 [0.64–2.16]	0.603		
ASA^a^ grading				
Ⅰ	Reference		Reference	
Ⅱ	1.18 [0.83–1.69]	0.348	1.04 [0.72, 1.50]	0.851
Ⅲ	2.26 [1.48–3.47]	<0.001	1.94 [1.24, 3.03]	0.003
Duration of anesthesia (hour)	1.03 [0.88–1.20]	0.707		
Duration of one-lung ventilation (hour)	0.92 [0.77–1.10]	0.385		
Anesthesia method				
INHA^a^	Reference			
TIVA^a^	1.08 [0.80–1.44]	0.623		
Regional blocks	0.74 [0.55–0.99]	0.044	0.71 [0.52, 0.96]	0.030
Morphine equivalent	1.00 [0.99–1.00]	0.231		
Vasoactive drugs	1.35 [1.01–1.80]	0.040	1.28 [0.95, 1.72]	0.104
Intraoperative hypoxemia	0.79 [0.22–2.84]	0.722		
Perioperative transfusion	1.42 [0.91–2.23]	0.122		
Infusion volume (mL)	1.00 [1.00–1.00]	0.656		
Urine volume (mL)	1.00 [1.00–1.00]	0.926		
Blood loss (mL)	1.00 [1.00–1.00]	0.515		
Surgical site (left)	1.01 [0.75–1.34]	0.961		
Type of operation				
Segmental/wedge resection	Reference			
Lobectomy	0.83 [0.61–1.12]	0.222		
Pneumonectomy	0.46 [0.05–3.90]	0.478		
VATS^a^	1.38 [0.94–2.03]	0.097		

Data are presented as the odds ratio [95% CI].

^a^ ASA, American Society of Anesthesiologists; BMI, body mass index; CI, confidence interval; INHA, inhalational anesthesia; TIVA, total intravenous anesthesia; VATS: video-assisted thoracic surgery.

In the total study cohort, univariate and multivariate logistic regression analyses were performed to explore the independent risk factors influencing PACU recovery time (Table 4). Multivariable analysis identified several independent risk factors: intraoperative dexmedetomidine administration (OR: 1.73; 95% CI: 1.34–2.23; *p* < 0.001), advanced age (OR: 1.02, 95% CI: 1.01–1.04; *p* = 0.005), smoking (OR: 1.52, 95% CI: 1.19–1.96; *p* = 0.001), cardiovascular disease (OR: 1.56, 95% CI: 1.07–2.23; *p* = 0.018), and ASA physical status III (OR: 1.90, 95% CI: 1.30–2.76; *p* = 0.001). Conversely, regional nerve block demonstrated a protective effect against prolonged PACU recovery time (OR: 0.76, 95% CI: 0.58–0.98; *p* = 0.035).

**TABLE 4:** Univariate and multivariate logistic regression analyses for PACU recovery time in the total study cohort.

Variable	Univariate logistic regression analysis		Multivariate logistic regression analysis	
	Odds ratio [95% CI^a^]	*p*-value	Odds ratio [95% CI^a^]	*p*-value
Dexmedetomidine				
NO-DEX	Reference		Reference	
DEX	1.68 [1.31–2.16]	<0.001	1.73 [1.34, 2.23]	<0.001
Age (years)	1.03 [1.01–1.04]	<0.001	1.02 [1.01, 1.04]	0.005
Sex (male)	0.81 [0.63–1.03]	0.081		
BMI^a^ (kg/m^2^)	1.01 [0.97–1.05]	0.719		
Smoking	1.54 [1.21–1.96]	<0.001	1.52 [1.19, 1.96]	0.001
Drinking	0.91 [0.70–1.19]	0.503		
Comorbidities at admission				
Hypertension	0.96 [0.71–1.28]	0.770		
Diabetes	0.96 [0.64–1.45]	0.852		
Cardiovascular disease	1.60 [1.13–2.26]	0.008	1.56 [1.07, 2.23]	0.018
Cerebrovascular diseases	0.95 [0.63–1.43]	0.810		
Respiratory diseases	1.01 [0.60–1.70]	0.974		
ASA^a^ grading				
Ⅰ	Reference		Reference	
Ⅱ	1.14 [0.85–1.53]	0.375	1.05 [0.78, 1.42]	0.751
Ⅲ	2.19 [1.52–3.14]	<0.001	1.90 [1.30, 2.76]	0.001
Duration of anesthesia (hour)	1.01 [0.89–1.15]	0.857		
Duration of one-lung ventilation (hour)	0.90 [0.78–1.05]	0.182		
Anesthesia method				
INHA^a^	Reference			
TIVA^a^	1.07 [0.83–1.37]	0.602		
Regional blocks	0.77 [0.06–0.99]	0.043	0.76 [0.58, 0.98]	0.035
Morphine equivalent	1.00 [0.99–1.00]	0.072		
Vasoactive drugs	1.19 [0.93–1.52]	0.159		
Intraoperative hypoxemia	0.83 [0.27–2.55]	0.750		
Perioperative transfusion	1.23 [0.83–1.82]	0.312		
Infusion volume (mL)	1.00 [1.00–1.00]	0.626		
Urine volume (mL)	1.00 [1.00–1.00]	0.854		
Blood loss (mL)	1.00 [1.00–1.00]	0.128		
Surgical site (left)	0.94 [0.74–1.20]	0.626		
Type of operation				
Segmental/wedge resection	Reference			
Lobectomy	0.81 [0.62–1.05]	0.113		
Pneumonectomy	0.23 [0.03–1.78]	0.159		
VATS^a^	1.20 [0.88–1.63]	0.246		

Data are presented as the odds ratio [95% CI].

^a^ ASA, American Society of Anesthesiologists; BMI, body mass index; CI, confidence interval; INHA, inhalational anesthesia; TIVA, total intravenous anesthesia; VATS: video-assisted thoracic surgery.

**FIGURE 4:**
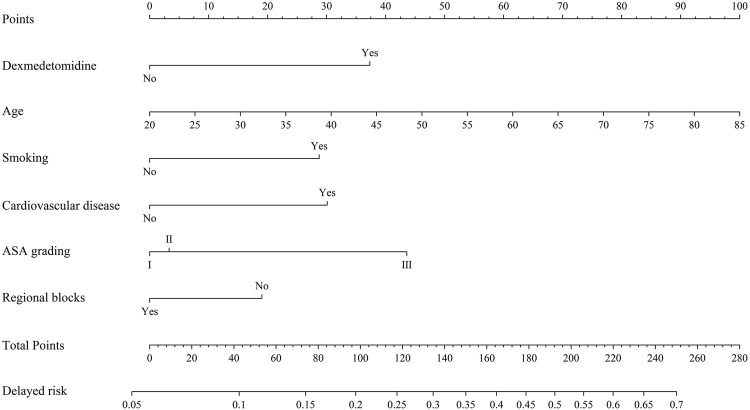
Nomogram for prediction of delayed recovery. Delayed risk means an incidence of delayed recovery after general anesthesia.

Based on the multivariate logistic regression analysis in the total study cohort, we developed a nomogram to delineate the contribution of risk factors to delayed recovery (Figure 4). This nomogram visually quantified each variable’s weight in the predictive model and estimated the probability of delayed recovery in the PACU. The results demonstrated that advanced age and ASA physical status III carried the highest point scores, contributing substantially to the increased likelihood of prolonged recovery. For patients presenting all risk factors, the predicted probability of delayed recovery in the PACU after surgery approached 70%.

**FIGURE 5:**
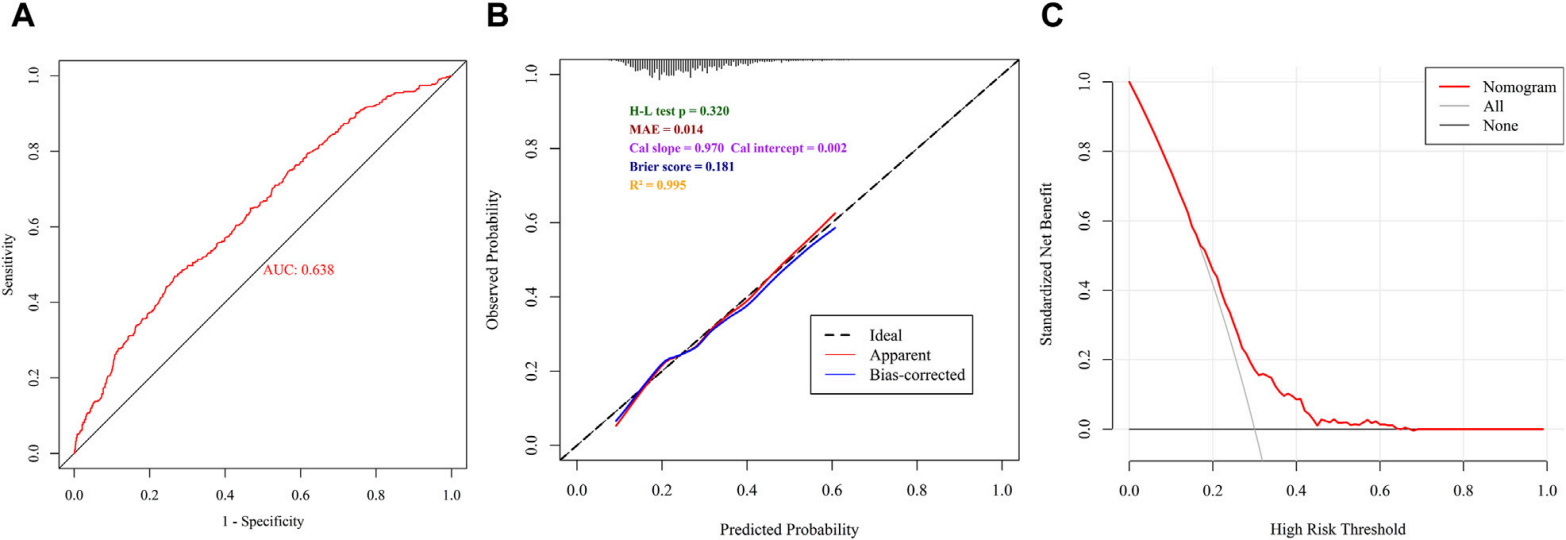
Performance of the nomogram. **(A)** ROC curve of the logistic regression model in the nomogram. **(B)** Calibration curve of the logistic regression model in the nomogram. **(C)** DCA of the logistic regression model in the nomogram.

The nomogram model predicting delayed PACU recovery exhibited moderate discrimination (AUC = 0.638, 95% CI: 0.605–0.671) (Figure 5A), although the calibration curve showed good agreement between predicted and observed outcomes (Figure 5B), and DCA demonstrated favorable clinical utility of the model (Figure 5C).

## Discussion

To the best of our knowledge, this investigation constituted the most extensive retrospective cohort analysis evaluating the relationship between intraoperative dexmedetomidine use and delayed recovery in the PACU following lung resection. The incidence of PACU recovery time exceeding 60 min was 25.48%. Intraoperative dexmedetomidine administration was associated with prolonged PACU recovery time. Sensitivity analyses under varying assumptions confirmed the robustness of our primary results. Independent risk factors for delayed recovery in the PACU included intraoperative dexmedetomidine use, advanced age, smoking, cardiovascular disease, and ASA physical status III, whereas regional nerve block was associated with reduced risk. The predictive nomogram demonstrated moderate discrimination and estimated a 70% probability of delayed PACU recovery in patients presenting with all identified risk factors.

Lung resection is the primary treatment for pulmonary diseases. However, noxious stimuli during general anesthesia, including endotracheal intubation, one-lung ventilation, extubation, and surgical tissue injury, can induce a significant stress response (Zhang B. et al., 2023; Gao et al., 2024). This leads to elevated concentrations of catecholamines, such as norepinephrine and epinephrine, in the blood, inducing hemodynamic fluctuations that eventually result in serious complications (Silpa et al., 2020; Miyazaki et al., 2022), including prolonged PACU recovery time (Gabriel et al., 2017; Zhang Q. et al., 2023). A large and retrospective cohort study found that among 38,796 patients undergoing general anesthesia, the average PACU recovery time was approximately 60 min (Zhang Q. et al., 2023). In addition, in another study, Feng et al. (2024) found that the average PACU recovery time after general anesthesia for patients undergoing thoracoscopic lung resection was approximately 51 min. In our study, the median PACU recovery time following lung resection under general anesthesia was approximately 50 min, with 25.48% of patients experiencing PACU recovery times exceeding 60 min, consistent with previous reports. Therefore, we need to identify the risk factors for delayed recovery in the PACU and improve its operational efficiency and medical cost savings.

Perioperative factors, such as preoperative comorbidities, ASA grade, age, anesthesia duration, intraoperative medication, type of surgery, and postoperative analgesia, may create a potentially adverse environment, leading to impaired wakefulness and recovery from general anesthesia, thereby prolonging the recovery time in the PACU (Zhang Q. et al., 2023). This adverse environment may be further aggravated by the use of anesthesia-related drugs that themselves have potential direct effects and indirect modulating effects on wakefulness and recovery under general anesthesia. Dexmedetomidine is a potent and highly selective α_2_-adrenoreceptor agonist. A number of prior studies demonstrated that dexmedetomidine, within an appropriate dose range, can effectively attenuate intraoperative stress response and postoperative pain, maintain perioperative hemodynamic stability, and decrease the incidence of adverse events, including emergence agitation, pain, opioid requirements, and nausea (Wu et al., 2023; Fu et al., 2024; Zheng et al., 2024; Zhu et al., 2024). In our study cohort, dexmedetomidine administration was also primarily indicated for perioperative sedation and anxiolysis to minimize other sedative medication usage. However, recent reports have suggested that these advantages are realized without delaying recovery (Sin et al., 2022). Other studies also indicate that dexmedetomidine prolonged early recovery in patients under general anesthesia (Adler et al., 2021; Huang et al., 2023). Our findings support this observation as patients in the DEX group demonstrated a 1.60-fold increased risk of delayed recovery in the PACU compared to those in the NO-DEX group. Moreover, we identified a significant relationship between dexmedetomidine doses and prolonged PACU recovery time, consistent with previous reports (Ma et al., 2021; West et al., 2021). This effect may be attributed to dexmedetomidine’s pharmacokinetic properties, particularly its elimination half-life of approximately 2–3 h and its potent sedative effects mediated through α_2_-adrenergic receptor agonism in the locus coeruleus.

This finding appears to contradict the established benefits of dexmedetomidine in promoting postoperative enhanced recovery. There are several possible explanations. First, most patients in our study received dexmedetomidine as a continuous intraoperative infusion rather than preoperative administration. Combined with dexmedetomidine’s longer half-life, this may have contributed to delayed PACU recovery. Second, dexmedetomidine has demonstrated a dose-dependent association with delayed PACU recovery (West et al., 2021). The relatively prolonged duration of lung resection resulted in an increased cumulative dexmedetomidine exposure, potentially contributing to delayed PACU recovery time. Third, our study did not maintain consistent anesthetic depth across patients as anesthesia was not titrated based on processed electroencephalogram monitoring. Consequently, patients receiving dexmedetomidine may not have received proportionally reduced doses of opioids and other sedatives, potentially contributing to oversedation and prolonged PACU recovery times. These findings warrant consideration by practitioners, who should recognize the potential for delayed recovery when weighing dexmedetomidine’s benefits. When incorporating dexmedetomidine as part of the anesthetic regimen, the dose of other central nervous system drugs should be reduced to prevent oversedation. Alternative agents (such as midazolam or remimazolam) may be considered substitutes for dexmedetomidine.

Prior research demonstrated that old age and ASA III lead to delayed recovery in the PACU (Zhang Q. et al., 2023), which was consistent with our findings. Tzabazis et al. (2015) further indicated that advanced age constituted a risk factor for delayed recovery. At first, delirium is arguably one of the most important postoperative complications because it is common, affecting up to 70% of patients older than 60 years undergoing major inpatient surgeries, and it is associated with adverse outcomes, including mortality, persistent cognitive decline, and delayed recovery from general anesthesia (Mashour et al., 2015; Horvath et al., 2021). Furthermore, slightly deeper anesthesia in vulnerable elderly patients with degenerative organ changes may increase the risk of postoperative delirium. Ultimately, the vulnerability of older patients and their susceptibility to postoperative delirium predispose them to delayed recovery in the PACU.

In addition, the severity of coexisting disease and the functional status of the patient are vital parameters for ASA grading before surgery (Horvath et al., 2021). The ASA grade was found to strongly correlate with outcomes and contribute to the assessment of risk and outcomes, at least with aggregate data (Kraev et al., 2018; Peters et al., 2020). Patients with an ASA grade of III have poor physical and organ function status, increasing their susceptibility to perioperative adverse events, resulting in delayed recovery from general anesthesia (Zhang Q. et al., 2023). Finally, the poor overall physical condition of the patients interferes with the metabolism and elimination of anesthetics in the body, and the resulting accumulation exceeding elimination rates may be a reason for delayed recovery. Similarly, we found that smoking and cardiovascular disease were associated with delayed recovery, which might be derived from their damage to the patient’s physical condition. Interestingly, we found that regional nerve block demonstrated a protective effect against delayed recovery, which may be attributed to its superior pain control and reduced opioid consumption.

This study had some limitations. First, this was a single-center retrospective observational study, with some limitations that cannot completely exclude residual confounding, despite propensity score matching being performed to reduce the potential confounding effect of each variable. Second, the retrospective design of this study limited our ability to obtain detailed information on the duration of continuous dexmedetomidine infusion, the timing of drug cessation relative to surgical completion, and the specific etiologies of delayed patient recovery, thereby restricting further causal analysis. Third, we must also admit that there was potential expectation bias among nursing staff in the PACU based on whether the patients had received dexmedetomidine during surgery: they may delay efforts to wake the patient in anticipation of a longer recovery. In addition, in patients receiving dexmedetomidine, there is a tendency to have the eyes closed even when the patients are awake. If nurses are unfamiliar with this effect of dexmedetomidine, it may lead to delayed discharge from the PACU. In each case included in the study, full compliance with the discharge criteria from the PACU was not confirmed, but we expect to follow the institutional guidelines described. In the future, all these factors need to be addressed in the large-sample and prospective cohort studies.

## Conclusion

In conclusion, this study demonstrated an association between intraoperative dexmedetomidine use and prolonged PACU recovery time following lung resection. The observed recovery delays may be of minor clinical significance, but their cumulative effect may create additional pressure on busy recovery rooms in hospitals with tightly regulated procedures. In addition, independent risk factors for delayed recovery in PACU included intraoperative dexmedetomidine use, advanced age, smoking, cardiovascular disease, and ASA physical status III, whereas regional nerve block was associated with reduced risk. Well-designed, prospective multicenter studies with large sample sizes and standardized dosing protocols are warranted to definitively establish the association between anesthesia-related medications such as dexmedetomidine and delayed recovery in the PACU.

## Data Availability

The raw data supporting the conclusions of this article will be made available by the authors, without undue reservation.
